# Optimization of iTRAQ labelling coupled to OFFGEL fractionation as a proteomic workflow to the analysis of microsomal proteins of *Medicago truncatula* roots

**DOI:** 10.1186/1477-5956-10-37

**Published:** 2012-06-06

**Authors:** Cosette Abdallah, Kjell Sergeant, Christelle Guillier, Eliane Dumas-Gaudot, Céline C Leclercq, Jenny Renaut

**Affiliations:** 1 Environmental and Agro-Biotechnologies Department, Centre de Recherche Public-Gabriel Lippmann, 41, rue du Brill, Belvaux, L-4422, Luxembourg; 2 UMR 1347 Agroécologie, CNRS, ERL CNRS 6300, BP 86510, Dijon, 21000, France; 3 UMR 1347 Agroécologie, INRA, BP 86510, Dijon, 21000, France

**Keywords:** Sample preparation, Membrane proteomics, Gel-free proteomics, OFFGEL peptide fractionation, iTRAQ labelling, Medicago truncatula

## Abstract

**Background:**

Shotgun proteomics represents an attractive technical framework for the study of membrane proteins that are generally difficult to resolve using two-dimensional gel electrophoresis. The use of iTRAQ, a set of amine-specific isobaric tags, is currently the labelling method of choice allowing multiplexing of up to eight samples and the relative quantification of multiple peptides for each protein. Recently the hyphenation of different separation techniques with mass spectrometry was used in the analysis of iTRAQ labelled samples. OFFGEL electrophoresis has proved its effectiveness in isoelectric point-based peptide and protein separation in solution. Here we describe the first application of iTRAQ-OFFGEL-LC-MS/MS on microsomal proteins from plant material. The investigation of the iTRAQ labelling effect on peptide electrofocusing in OFFGEL fractionator was carried out on *Medicago truncatula* membrane protein digests.

**Results:**

In-filter protein digestion, with easy recovery of a peptide fraction compatible with iTRAQ labelling, was successfully used in this study. The focusing quality in OFFGEL electrophoresis was maintained for iTRAQ labelled peptides with a higher than expected number of identified peptides in basic OFFGEL-fractions. We furthermore observed, by comparing the isoelectric point (pI) fractionation of unlabelled versus labelled samples, a non-negligible pI shifts mainly to higher values.

**Conclusions:**

The present work describes a feasible and novel protocol for in-solution protein digestion in which the filter unit permits protein retention and buffer removal. The data demonstrates an impact of iTRAQ labelling on peptide electrofocusing behaviour in OFFGEL fractionation compared to their native counterpart by the induction of a substantial, generally basic pI shift. Explanations for the occasionally observed acidic shifts are likewise presented.

## Background

Two-dimensional gel electrophoresis (2-DE) coupled to mass spectrometry (MS) has been the trademark method for relative protein quantification in plant proteomics. Nevertheless, lack of quantitative reproducibility [1], poor representation of low abundant proteins, highly acidic/basic proteins [2], or proteins with extreme size or hydrophobicity [3] are the principal shortcomings of 2-DE, this related to the low tolerance of the technique for detergents and ionic compounds. However, handling membrane proteins requires detergent and buffer use for membrane solubilisation and homogenization followed by their removal prior to further analysis in MS. Proteomic analysis of membrane proteins remains a major challenge and represents an ongoing topic of myriad investigations. Therefore, MS-based 2D-gel-free proteomic approaches have recently bypassed the status of descriptive tool to become the new mainstream method for quantitative proteome studies.

Isobaric tags for relative and absolute quantitation (iTRAQ) are recently developed chemical labelling reagents [4] that quickly gained popularity in proteomics [5,6]. The iTRAQ label modifies peptide N-termini and ϵ-amino groups of lysine side chains. It was shown to increase the number of peptides identified by MS, a finding attributed to a greater number of lysine-terminated peptides detected [7]. Protein quantification relies on reporter ions generated in a “silent region” at low molecular mass of peptide MS/MS spectra. iTRAQ labelling proved compatibility with different kind of samples, providing in-depth knowledge in several biological pathways and has been applied in plant shotgun proteomics [8]. Majeran and co-workers used iTRAQ for a comparative analysis of the chloroplast envelope proteome in maize [9], and the approach was likewise used to study the changes in the *Arabidopsis* plasma membrane in response to flagellin treatment [10].

Immobilized pH gradient isoelectric focusing (IPG-IEF) has emerged as a highly promising alternative to strong-cation exchange fractionation as the first separation dimension in shotgun proteomics [11], especially for membrane proteome analysis [12,13]. OFFGEL electrophoresis (OGE) combines the traditional IEF using IPG strips with the convenience of a liquid-based system. Proteins or peptides migrate through the IPG strip until they reach their isoelectric point (pI) at a given compartment, and after completion of the run samples can be easily recovered in solution for further analysis. OGE separation as first step was recently compared to MudPIT for the analysis of membrane proteins and resulted in comparable results for protein/peptide identification and reproducibility [14]. The inclusion of OGE into the proteomic workflow furthermore offers the opportunity to determine the pI of peptides which is an independent validating and filtering tool for false positive identifications [15]. The additional use of a pI filter enhances the stringency of the peptide validation criteria and increases the identification confidence. However, most frequently used pI calculation algorithms use only native peptide sequences, and the addition of modifications such as the iTRAQ label is cumbersome. The question whether an iTRAQ labelled peptide will exhibit the same pI-value as the native counterpart is therefore not trivial. In the present study, an online tool for chemical drawing, MarvinSketch calculator (http://www.chemaxon.com/marvin/sketch) has been used to calculate pI of unlabelled and iTRAQ labelled peptides to explain some experimentally observed pI shifts [16].

A quantitative proteomic approach using iTRAQ-IEF combination was successfully applied on *Staphylococcus aureus* membrane extracts [17]. Moreover, Chenau and co-authors evaluated the efficiency of OGE fractionation for iTRAQ labelled peptides from the human secretome and plasma [18]. When evaluating OGE fractionation of iTRAQ labelled peptides, one must consider that the iTRAQ-label incorporates a highly basic group “*N*-methylpiperazine” at peptide N-termini and ϵ-amino groups of lysine side chains. This can alter the pI of peptides and consequently the isoelectrofocusing behaviour in IPG-IEF or OGE. This impact was previously studied using proteins from a colon cancer cell line using a small, acidic pH range between pI 3.4 and 4.9; and there any observed shift in pI could only be small or absent [19].

To date, the iTRAQ/OGE couple has been applied on complex eukaryotic samples and different types of matrices, but none dealing with plant membrane proteins [18,20]. Here we present, for the first time, the application of an iTRAQ-OGE-LC-MS/MS proteomic approach on microsomal proteins from *Medicago truncatula* roots. A feasible protocol is described for in-solution protein digestion allowing the recovery of a “clean” protein digest from *Medicago truncatula* cv Jemalong 5 roots inoculated or not with *Rhizophagus irregularis.* Furthermore by comparing the OGE fractionation of native and labelled peptides, the predictable basic shift induced by iTRAQ labelling was studied using a wide pH-range (3-10).

## Results and discussion

### Experimental design

**Figure 1:**
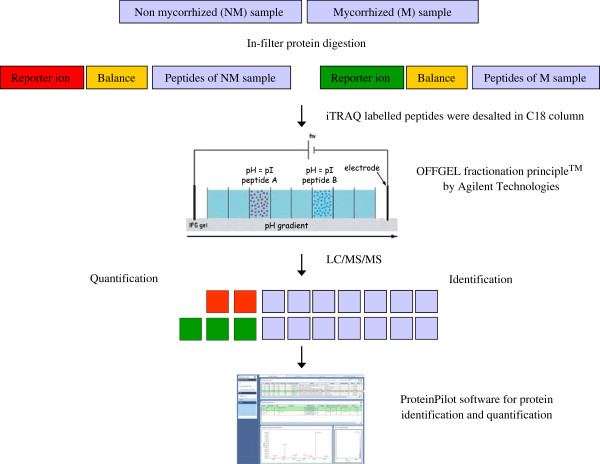
**Schematic representation of the experimental workflow of iTRAQ-OGE-LC-MS/MS.** The flowchart shows the peptide OGE fractionation process designed by Agilent Technologies [22].

During the last decade, proteomics has gained popularity in plant science, but still mostly relies on 2-DE. Not all types of proteins are amenable to gels and this method often falls short to study low-abundant and recalcitrant proteins. Therefore a gel-free proteomic approach was implemented here on *Medicago truncatula* membrane proteome. For microsome preparation a previously optimised method based on differential centrifugation has been employed [21]. Microsomal proteins were cleaved using a homemade protocol for in-solution protein digestion allowing the recovery of a “clean” peptide fraction. Subsequently, these protein digests from *M. truncatula* roots inoculated or not with *Rhizophagus irregularis*, were labelled with iTRAQ and fractionated using OGE prior to RP-HPLC-MS/MS, a first time this type of approach is used on membrane proteins of plant material. OGE prefractionation was performed in 12 wells using a 12 cm strip covering the pH range of 3 to10. iTRAQ labelled and pre-fractionated samples were then separated using liquid chromatography (LC) followed by MALDI-TOF/TOF analysis. Searches in the databases were carried out using ProteinPilot software. A schematic summary of the workflow performed in the current study is illustrated in Figure 1.

The OGE-LC separation of 100 μg of unlabelled microsomal protein digest allowed the identification of 241 peptides and 107 proteins, whilst 266 peptides and 130 proteins were identified in iTRAQ labelled samples. The enrichment of membrane protein fraction was assessed by the sub-cellular localisation of the identified proteins. Seventy percent of proteins in both experiments had at least one membrane localisation experimentally demonstrated (results not shown). Furthermore only 7 and 5% of the identified proteins were predicted to be localised in the cytosol in unlabelled and iTRAQ labelled experiments, respectively (results not shown).

### In-filter protein digestion

**Figure 2:**
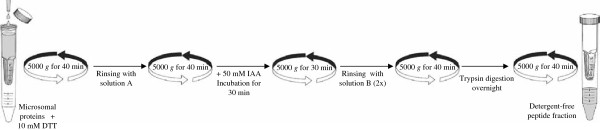
**Depiction of in-filter protein digestion protocol using Amicon Ultra filter devices**^**TM**^**(Millipore) **[27].

One of the most critical steps in all proteome analyses is sample preparation. Detergents are indispensable tools for the solubilisation and fractionation of membrane proteins. However, they can dominate mass spectra and preclude peptide analysis in MS [23], even in minute concentrations. As a consequence, the majority of studies on membrane proteins use in-gel digestion to remove detergents prior to mass spectrometric analysis [24]. Therefore, gel-free proteomic approaches require adequate protocols for in-solution protein digestion while avoiding the use of high-ionic strength buffers and detergents. To overcome these difficulties, various alternative approaches have been described and the use of filtration columns appears to be the most promising [25]. Based on the filter-aided sample preparation (FASP) workflow [26], a method has been developed in the current study in which the protein digestion took place in a commercially available ultra-filtration device used for protein retention, buffer exchange and removal (Figure 2). The key feature of this method is the ability to remove interfering compounds associated with the sample during protein digestion through the filter device and to recover resulting peptides by centrifugation. Wiśniewski and co-workers compared the distribution of molecular weights of the identified proteins using either a 3k or 10k filter. They found that the 10k filter efficiently retained small proteins (5-10kDa) and efficiently released peptides up to 5,000 Da [26]. Therefore, Amicon Ultra filter devices (Millipore), with relative molecular mass cut-off of 10,000 NMWL (Nominal Molecular Weight Limit) have been used in the subsequent experiment. The in-solution protein digestion protocol was made up of 3 main steps: (1) DTT was first added to reduce protein disulphide bonds. Then, (2) carbamidomethylation of thiols was achieved by the addition of iodoacetamide. All the reagents added during these steps were easily removed by centrifugation. Afterwards, (3) protein digestion was carried out by adding trypsin and leaving it overnight at room temperature. Finally, the peptide fraction, free of unwanted, interfering compounds, was obtained by centrifugation.

One of the aims of the current study was to develop an alternative in-solution protein digestion to the one proposed by iTRAQ reagent kit in order to be able to produce peptide fraction free of interfering compounds such as urea, Tris and DTT. Consequently, in-filter protein digestion represented the method of choice allowing the removal of residual interfering buffers associated with the sample. At one step to iTRAQ labelling, any buffer added through sample preparation should be “primary amine free” to avoid the quenching of the iTRAQ labelling. Hence, ammonium bicarbonate’s substitution was a mandatory step. Triethylammonium bicarbonate (TEAB) was chosen as a good tertiary amine buffer, which is also very volatile, and can therefore be easily removed *in vacuum*. Thus in our protocol, the in-filter protein digestion described above was convenient for protein digestion and sample clean-up prior to iTRAQ labelling.

### Peptide isobaric tagging

After the labelling process, samples must be ready for OGE separation. Nevertheless, excess iTRAQ reagents in the sample mixture should be removed since their presence can suppress the signal obtained from target peptides and thus reduces the level of achievable sensitivity and reproducibility. The supplier recommended the use of cation exchange (CX) cartridge, delivered with the iTRAQ kit, as a desalting step prior to LC-MS/MS analysis. However since peptides will be eluted of the CX-column in 10 mM potassium phosphate in 25% (v/v) of acetonitrile (ACN) and 350 mM of potassium chloride, when using the OGE fractionator, an alternative desalting method was an absolute requirement. Several tests with alternative desalting steps have been conducted, and it was found that the use of a C18 column was well suited to clean-up iTRAQ labelled samples (results not shown). Ernoult and co-authors have also used C18 cartridge to desalt the iTRAQ labelled samples prior to OGE [7].

**Figure 3:**
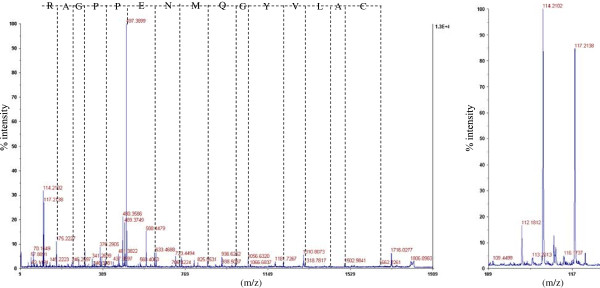
**Spectrum of an iTRAQ labelled peptide of control (114) and mycorrhized (117) protein digest.** (A) MS/MS spectrum of iTRAQ labelled CALVYGQMNEPPGAR at m/z 1806.94. (B) iTRAQ reporter ions at m/z 114 and 117, their peak areas are used to calculate the relative abundance of a given peptide.

Since we have used an alternative in-solution digestion protocol, which contains DTT, IAA and Tris categorized as potential interfering substances with the labelling process, the utility of this method for the removal of these compounds and thus the creation of an iTRAQ-compatible environment needed to be established. One example of a labelled peptide is shown in Figure 3. Figure 3A presents the MS/MS spectrum of CALVYGQMNEPPGAR at m/z 1806.94, while 3B shows the low mass region covering daughter ions (114 and 117) released in MS/MS. Further empirical evidence that the applied procedure was successful in removing interfering compounds was obtained by researching all datasets but omitting the fixed modification with the iTRAQ label (not added or added as variable modification). None of these searches resulted in the significant identification of a peptide, so if not all interfering compounds are completely eliminated at least their effect on labelling was not observable in our data.

### Peptide OGE fractionation

IPG as first dimension separation strategy has proved to be superior to SCX with a salt or pH gradient [28,29]. Therefore, OGE has been chosen to separate peptides according to their isoelectric point in a liquid phase. The novelty and strengths of this method can be resumed by the ability to directly introduce pI-fractionated peptides to LC-MS/MS analysis. Nonetheless, glycerol in peptide focusing buffer interfered with SpeedVac concentration (increased viscosity), direct injection into reverse phase LC and with crystallization on a MALDI target. This problem was previously mentioned by Fraterman and co-workers [30], in which study the glycerol content of the focusing buffer was reduced by 50% (v/v) in deviation from the supplier’s protocol, while others indicated the use of even lower concentrations [31,32]. Several other studies did not mention the concentration of glycerol proposed by the OGE manufacturers (6%) as a problem [7,18]. In the present work, reducing the concentration of glycerol by 50% (v/v), final concentration equals 3%, was not enough to avoid the clogging of the pre-column after few runs or to solve the crystallization problem on a MALDI target. According to Agilent Technologies, reducing and even omitting glycerol content in peptide focusing buffer does not affect the efficiency of the IEF, therefore the glycerol concentration was reduced to 5% (v/v) (final concentration equals 0.3%) in deviation of the original protocol. Hubner and co-workers demonstrated that the loading capacity for optimal peptide focusing on 12 cm strip is below 100 μg [31,32], therefore in this study, 100 μg of protein digest were separated in OGE. The isoelectrofocusing of peptides offers the possibility to exploit the deviation between expected and observed peptide pI distribution across the IPG strip. It has been reported that the average pI values of peptides fits fairly well with the pH range of the corresponding OGE fractions [7,15,18].

**Figure 4:**
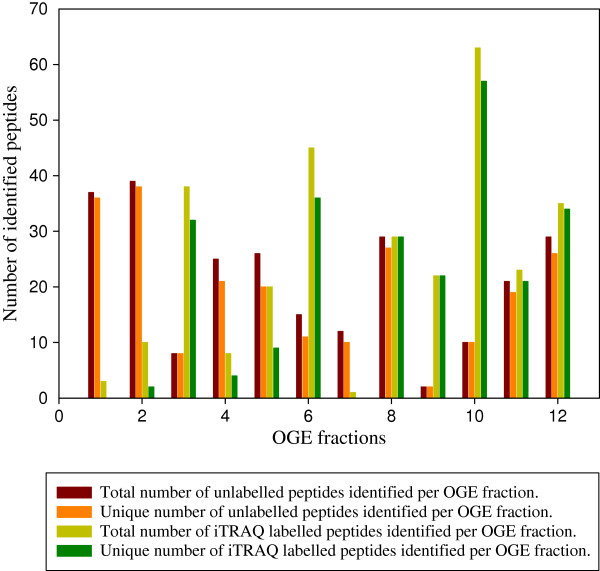
**Number of peptides identified per OGE fraction.** Brown and light green bars represent the total number of unlabelled and iTRAQ labelled peptides identified in each fraction, respectively. Orange and dark green bars indicate the unique number of unlabelled and iTRAQ labelled peptides identified per fraction, respectively.

Hence, the effective resolution obtained in the 12 OGE fractions of free and iTRAQ labelled samples was assessed by determining the number of peptides identified in single versus multiple fractions (Figure 4). Peptides were unevenly distributed along the IPG strip in both labelled and unlabelled samples. In native samples, over 70% of identified peptides were localized in only one fraction and more than 90% were found in one or two successive fractions. These findings were in agreement with previous studies [15]. In iTRAQ labelled samples, more peptides were identified in basic region compared to the acidic one. Only 3, 10 and 8 peptides in total were respectively found in fraction 1, 2 and 4 while 63 peptides were identified in fraction 10. Moreover, the fractionation quality in basic fractions was greater than in acidic ones. More than 80% of peptides were recovered in single fraction (fractions 6 to 12) while this percentage fell to 20% in fraction 2 and no unique peptide was found in fraction 1. As Ernoult *et al.* (2008) have shown, more peptides were recorded in iTRAQ labelled samples giving the fact that iTRAQ improves MALDI ionisation [7,15,18]. Our results confirmed that the slightly modified version of the initial OGE protocol applied did not affect the quality of peptide IEF. Interestingly, iTRAQ labelled peptides showed a better OGE fractionation quality in basic fractions where a greater number of peptides have been identified compared to acidic ones. This observation will be discussed in more detail below.

### iTRAQ impact on peptide OGE fractionation

The assumption that iTRAQ labelling induces a negligible increase in peptide isoelectric point [19] prompted us to investigate the validity of this claim on peptide OGE fractionation on a wide pH range (3-10). Hence, peptide distribution on the 12 cm strip pH 3-10 was examined when peptides were either labelled or not with iTRAQ reagents. The current survey delineated 4 different groups of labelled peptides found in at least 3 replicates of labelled samples with a high reproducibility in these independent experiments (Additional file 1).

Table 1 shows a set of 70 peptides (group A) found in more basic fractions after iTRAQ labelling, this finding being highly related to the incorporation of the basic group “*N*-methylpiperazine” at peptide N-termini and ϵ-amino groups of lysine side chains. As an example, 2 ions were assigned to EQMGYTFDALK by Paragon search with 99% of confidence. The unlabelled peptide with a m/z of 1301.58 was found in fraction 1 while the labelled (m/z 1590.80) was identified in fraction 3. The mass difference corresponds 289.22 Da or the addition of two labels at the N-terminus and the ϵ-amino groups of lysine side chain. For peptides only containing one label at the N-terminus the basic shift was, as previously described by Ross and co-workers, in general more important [4]. For instance NYTNAFQALYR (m/z 1504.76), exhibiting only one potential iTRAQ modification site, the labelled form of this peptide was found in fraction 12 while its native counterpart at m/z 1359.65 was focused in fraction 10. Most of the peptides in group A followed the same trend with a shift of 1 to 2 fractions to the basic end of the strip (Table 1, group A). Some others (7 peptides) pursued the observed basic shift tendency but are focussed irreproducible using the OGE fractionator (Additional file 1, group D).

**Table 1:** Observed basic pI shifts in OGE fractionation after iTRAQ labelling

	Sequence	Mass NL	Mass L	Difference	F NL	F L
1	ECADLWPR	1045.46	1190.57	145.11	1	3,4
2	AYLEDFYR	1075.50	1220.61	145.11	1	3,4
3	EQMGYTFDALK	1301.58	1590.81	289.23	1	3,4
4	TMADEGVVALWR	1346.65	1491.77	145.12	1,2	3,4
5	ECSGVEPQLWAR	1430.66	1575.77	145.11	1	3,4
6	NQIDEIVLVGGSTR	1499.78	1644.90	145.13	1	3,4
7	LAEMPADSGYPAYLAAR	1794.84	1939.97	145.13	1	3,4
8	ELEFYMK	958.46	1247.66	289.20	1	3
9	IPSAVGYQPTLSTDLGGLQER	2201.13	2346.24	145.11	1	3,4
10	AQIWDTAGQER	1273.59	1418.71	145.12	1	3,4
11	AGGECLTFDQLALR	1549.77	1694.87	145.10	1	3,4
12	VDFAYSFFEK	1251.61	1540.79	289.18	1	3
13	IFDKPEDFIAER	1478.74	1767.95	289.22	1	3,4
14	GLFTSDQILFTDTR	1612.79	1757.92	145.13	1	3,4
15	TTPSYVAFTDSER	1472.66	1617.79	145.13	1	3,4
16	LDTGNFSWGSEAVTR	1638.76	1783.87	145.11	1	3,4
17	LWQVPETLPAEVVGK	1664.93	1954.13	289.19	2	3,4
18	QLDAHIEEQFGGGR	1555.73	1700.85	145.11	2	3,4
19	GFGFVTFAEEK	1230.61	1519.80	289.19	2	3,4
20	AFLVEEQK	962.51	1251.72	289.21	2	3,4
21	IFEGEALLR	1046.59	1191.69	145.10	2	3,4
22	ISGLIYEETR	1179.61	1324.72	145.11	2	3,4
23	TTAEEGVVALWR	1330.71	1475.80	145.09	2	3,4
24	LLIQNQDEMIK	1343.72	1632.92	289.21	2	3,4
25	IQDKEGIPPDQQR	1522.77	1811.99	289.22	2	3,4
26	TMVYPEAGFELQR	1539.74	1684.85	145.10	2	3,4
27	EQDVSLGANKFPER	1588.77	1878.00	289.22	2	3,4
28	GQGGIQQLLAAEQEAQR	1795.93	1941.03	145.10	2	3,4
29	QYAVFDEK^$^	981.46	1287.68	306.22	2	3,4
30	QLDSHIEEQFGGGR^$^	1554.71	1716.84	162.13	2	3,4
31	FDVGVKEIEGWTAR	1605.84	1895.03	289.19	2	3,4
32	HFEVDLSAFR	1219.61	1364.71	145.10	3	4
33	CALVYGQMNEPPGAR	1661.78	1806.87	145.10	4	6
34	NAVVTVPAYFNDSQR	1679.84	1824.94	145.10	4	5,6
35	QPTELELAQAFHQGK	1695.85	1985.07	289.22	4	5
36	TALTYVDNNDGSWHR	1747.80	1892.90	145.10	4	5
37	QQFPLALYQVDK	1448.78	1737.98	289.20	4	5,6
38	ADGFAGVFPEHK	1273.60	1562.82	289.22	4	5
39	SLEGLQANVQR	1213.63	1358.75	145.13	4	6
40	TFDNVYYK	1048.47	1337.70	289.23	4	5
41	MLSPLILGDEHYQTAR	1842.96	1988.04	145.08	4	5
42	SSFDAFQQILK	1282.67	1571.87	289.20	4	5
43	SSDFLMYGIK	1159.57	1448.77	289.20	4	5
44	SSMDAFQQILK	1282.67	1571.83	289.17	5	5
45	FTQANSEVSALLGR	1491.77	1636.88	145.11	5	6
46	STLVWEVR	988.56	1133.64	145.08	5	6
47	IFLENVIR	1002.62	1147.70	145.07	5	6
48	FFCEFCGK	1093.48	1382.65	289.17	5	6
49	EVAGFAPYEKR	1265.65	1554.85	289.20	5,6	6
50	SFGPAVIFNNEK	1321.70	1610.88	289.18	5	6
51	VALINYGPEYGR	1350.75	1495.80	145.06	5	6
52	YIAPEQVPVK	1142.63	1431.85	289.21	5	5,6
53	VEPLVNMGQITR	1355.72	1500.83	145.12	5	6
54	AYEPILLLGR	1143.67	1288.77	145.10	5	6
55	LVGEYGLR	905.51	1050.61	145.10	5	6
56	EALGGLPLYQR	1215.66	1360.77	145.11	5	6
57	ADAFLLVGTQPR	1286.70	1431.81	145.10	5	6,7
58	HGWEYVVK	1016.51	1305.72	289.21	6	8
59	FVIGGPHGDAGLTGR	1452.75	1597.86	145.10	7	8
60	LVNVFTIGK	989.60	1278.80	289.20	7	9,10
61	THAVVEPFVIATNR	1552.86	1697.95	145.09	7	8,9
62	SVHEPMQTGLK	1225.62	1514.82	289.21	7	8
63	SVVYALSPFQQK	1365.74	1654.94	289.20	7	10
64	YGGGANFVHDGYNK	1497.61	1786.88	289.27	7	8
65	TALTYIDGNGNWHR	1616.76	1761.88	145.12	8	9
66	AHLQDYIQTHYTAPR	1812.89	1958.00	145.11	8	9
67	NYTNAFQALYR	1359.66	1504.77	145.11	10	12
68	TLHPNWSPAAIK	1333.72	1622.93	289.21	10	11
69	FHQYQVVGR	1132.56	1277.69	145.13	11	12
70	FQSLGVAFYR	1186.64	1331.72	145.08	11	12

This table presents 70 peptides shifted to more basic OGE fraction after iTRAQ labelling. The non-labelled (NL) and iTRAQ labelled (L) peptide masses are shown in this table together with the mass difference induced by the labelling. The OGE fractions (F) of iTRAQ labelled peptides and their native counterparts are as well presented.

As shown in Table 2, 34 peptides were categorized in group B where peptides in both label-free and iTRAQ labelled experiments are focussed in the same OGE fraction. Among them, 10 peptides were recovered in fraction 12 the most basic fraction. Hence, even if iTRAQ labelling rendered them more basic, fraction 12 stands for the last and most basic region of the 12 cm strip (pH 3-10) a peptide could reach. Furthermore, 10 of the 34 peptides were discerned (marked with an asterisk * in Table 2) to register the expected basic shift in at least one experiment but this shift could not be observed in all replicates. The fourteen remaining peptides were focused in the same OGE fraction before and after iTRAQ labelling. The most reliable explanation to this observation was that the iTRAQ tag did induce an increase in the peptide pI, but this shift was not pronounced enough and therefore did not provoke a shift in fraction since every fraction corresponds to 0.6 pH-unit. To look into this further, peptide amino acid structures were manually drawn in MarvinSketch calculator and peptide isoelectric points were calculated for the native and iTRAQ modified peptides. For example, the non-modified peptide GFGFVTFANEK of m/z 1215.62 was found in the same OGE fraction (fraction 6) of its doubly labelled peptide of m/z 1504.80. The calculated pI of the unlabelled peptide is 5.94 while a pI of 5.97 is assigned to the iTRAQ labelled form. Thus, the pI was slightly modified after iTRAQ labelling and remained below the edge of 0.6 pH-units.

**Table 2:** Co-migration of iTRAQ- and unlabelled peptides in the same OGE fraction

	Sequence	Mass NL	Mass L	Difference	F NL	F L
1	MFDAGLYEHCR*	1397.58	1542.69	145.12	4	4
2	EAFPGDVFYLHSR*	1536.75	1681.85	145.09	4	5
3	TLHGLQPPESSGIFNEK*	1852.93	2142.14	289.22	4	4
4	ATFDCLMK*	984.49	1273.65	289.16	5	5
5	GIPYLNTYDGR*	1267.67	1412.73	145.06	5,6	5,6
6	GFGFVTFANEK	1215.62	1504.80	289.18	2,6	6
7	GKDFAELIASGR	1262.67	1551.87	289.21	6	6
8	AALNDFDRFK	1195.60	1484.81	289.21	6	6
9	EAQWAHAQR*	1095.52	1240.63	145.11	8	8
10	SRFFHSTGQR*	1221.54	1366.71	145.17	8	8
11	AGDFFHSAQSR*	1221.56	1366.66	145.11	8	8
12	ASALIQHDWSR*	1282.63	1427.75	145.12	8	8
13	AHGGFSVFAGVGER	1389.69	1534.79	145.10	8	8
14	VGPFHNPSETYR	1402.65	1547.77	145.12	8	8
15	GVDKEHVMLLAAR	1437.77	1726.99	289.22	8	8
16	EVHFLPFNPVDKR	1596.85	1886.05	289.20	8	8
17	EIHFLPFNPVDKR	1610.87	1900.07	289.20	8	8
18	ALYHDLNAYR	1234.61	1379.72	145.11	8	8
19	AGVKPHELVF	1095.61	1384.82	289.21	8	8
20	LAWHSAGTFDSK*	1318.61	1607.84	289.23	8	8
21	YDTVHGQWK	1132.52	1421.74	289.22	8	8
22	NGGANFVAPGYTK	1294.54	1583.84	289.31	10	10
23	AASFNIIPSSTGAAK	1433.76	1722.96	289.21	10	10
24	FVTAVVGFGK	1023.57	1312.79	289.21	10	10
25	AGQYNFLIR	1080.58	1225.68	145.10	12	12
26	AYGGVLSGGAVR	1105.59	1250.70	145.10	12	12
27	GFQTSYYNR	1134.50	1279.62	145.12	11,12	12
28	VLNTGSPISVPVGR	1394.76	1539.90	145.13	12	12
29	SSSVFIPHGPGAVR	1409.73	1554.85	145.12	12	12
30	LVSAHSSQQIYTR	1488.75	1633.88	145.13	12	12
31	GGGHTSQIYAIR	1258.65	1403.75	145.11	12	12
32	HGSLGFLPR	982.53	1127.64	145.12	12	12
33	GGQLIYGGPLGR	1186.63	1331.76	145.12	12	12
34	AGGAYTLNTASAVTVR	1550.79	1695.91	145.12	12	12

Thirty-four peptides focused in the same OGE fraction in both label-free and iTRAQ labelled experiments are shown in this table. Peptides shifted to more basic fractions in at least one experiment are indicated with an asterisk (*).

Table 3 shows the eleven peptides of group C that were focused in more acidic fractions when they were iTRAQ labelled. Initially, the native form of these peptides was found in fractions 11 and 12, gathering the most basic peptides. The observed acidic shift is furthermore in agreement with Chenau *et al.* (2008). These authors found that the peptide pI average in fraction 24 (most basic fraction in their experiment, strip pH 3-10, 24 cm) decreased from 9.22 to 8.74 in non-labelled and iTRAQ labelled samples, respectively [18]. To further investigate the acidic shift induced by iTRAQ labels, MarvinSketch Calculator was again implemented for pI calculations. The calculated pI of SKFDNLYGCR is 8.32 and it decreases to 7.72 after labelling, which corresponds to a shift of one OGE fraction. Indeed, the non-modified peptide was focused in fraction 11 while its iTRAQ labelled form was retrieved in fraction 10. Moreover, ASALIQHEWRPK, focused in fraction 12 when non-modified, was found in a more acidic fraction (fraction 10) after iTRAQ labelling. Interestingly, the calculated pI of the native peptide is 9.12 while this is 7.04 for its doubly labelled form, which corresponds accurately to the experimentally observed pI shifts.

**Table 3:** Acidic pI shifts in OGE as a result of iTRAQ labelling

	Sequence	Mass NL	Mass L	Difference	F NL	F L
1	SKFDNLYGCR	1258.56	1547.79	289.22	11	10
2	QFNGLVDVYKK^$^	1292.69	1743.02	450.33	11	9,10
3	KGPLIVYGTEGAK	1331.73	1765.06	433.33	11	9
4	YLQPQESGWKPK	1459.80	1893.06	433.26	11	9,10
5	GVQQVLQNYK	1175.61	1464.84	289.23	12	10
6	KQFVIDVLHPGR	1407.81	1697.01	289.20	12	10
7	ASALIQHEWRPK	1434.76	1723.98	289.22	12	10
8	LSEPYKGIGDCFKR	1668.82	2102.14	433.33	12	9,10
9	GVLPQNQPFVVK	1324.74	1613.96	289.22	12	10,11
10	INWLTNPVHK	1220.65	1509.88	289.22	12	10
11	IAGFSTHLMK	1103.57	1392.79	289.22	12	10

Eleven peptides shifted to more acidic OGE fraction after iTRAQ labelling are presented in this table.

^$^Gln- > pyro-Glu@N-term.

### Acidic and basic amino acid distribution per peptide

**Figure 5:**
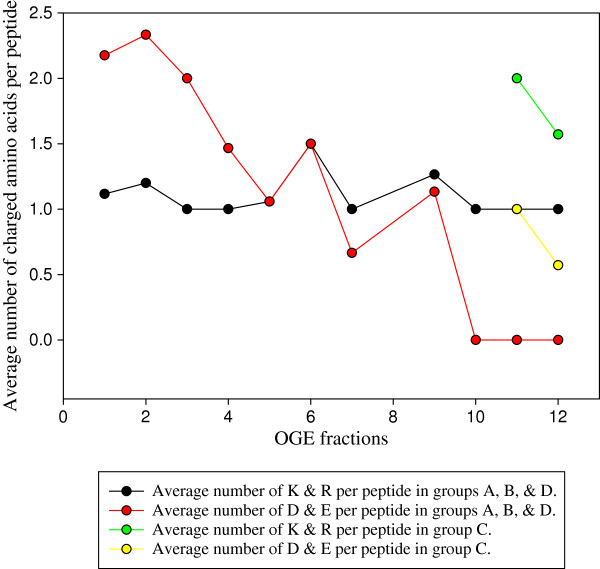
**Average distribution of basic and acidic amino acids per peptide in each OGE fraction.** Average number of acidic (red plot) and basic (black plot) amino acids per peptide in groups A, B and D. Yellow and Green plots correspond to the average number of acidic and basic amino acids per peptide in OGE fractions 11 and 12 in group C, respectively.

Some molecular considerations have been introduced to explain the behaviour of the 111 peptides in groups A, B and D presenting the expected basic shift and the 11 peptides in group C showing an acidic shift after iTRAQ labelling. Peptide amino acid composition has been investigated with a special attention to the acidic (E and D) and basic (K and R) amino acids. Thus, the average number of each amino acid by peptide per fraction was calculated (Figure 5). In peptides that shifted to basic regions (groups A, B and D), the number of acidic amino acids D and E by peptide globally decreased from acidic to more basic fractions to be absent in fraction 10 and above, a finding in agreement with Chenau *et al.* (2008) [18]. Contrary to this, 7 of the 11 peptides that shifted to a more acidic fraction after labelling (Table 3, group C) contain at least one acidic residue and were nonetheless found in the fractions 11 and 12 when not labelled. Furthermore, the number of basic amino acids is higher in peptides that have an acidic shift compared to peptides that have a basic shift (Figure 5). Exclusively in the group showing acidic shifts, 4 peptides (KGPLIVYGTEGAK, QFNGLVDVYKK, YLQPQESGWKPK and LSEPYKGIGDCFKR) of the 11 had 3 iTRAQ tags due to the presence of two lysines in their sequences while no similar case has been observed in peptides shifting to more basic fractions. The experimental finding that iTRAQ labelling shifts the pI of lysine-containing peptides to more acidic values was corroborated by pI calculations in MarvinSketch Calculator on the different forms of lysine. The calculated pI of native, free lysine (9.82) decreases to 7.60 for the double-tagged amino acid.

### iTRAQ impact on peptide elution time

**Figure 6:**
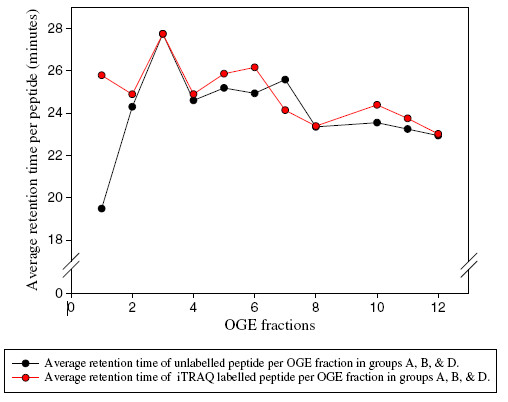
**Peptide elution time in LC separation.** Average retention time (in minutes) of iTRAQ labelled peptides (red plot) and their native counterparts (black plot) in each OGE fraction in groups A, B and D.

The degree of retention time variation in LC separation after iTRAQ labelling was examined in groups A, B and D. Resulting retention time values were plotted per fraction for the unlabelled and iTRAQ labelled samples as shown in Figure 6. Clearly, iTRAQ labelling increased the retention time of 17 peptides initially focused in OGE fraction 1 and shifted to more basic fractions after the iTRAQ modification by approximately 6 minutes (Additional file 2). This variation decreased to less than 1 minute in other OGE fractions, to be entirely gone in fractions 3, 4, 8 and 12 where iTRAQ labelled peptides and their native counterparts eluted at approximately the same time. For group C, label-free and iTRAQ labelled peptides recorded almost the same elution time in LC separation (results not shown). Thus, iTRAQ labelling drastically affected peptide retention time in OGE fraction 1 and lost its effect for the more basic fractions.

## Conclusions

In plants, MS-based proteomics has been largely used for protein identification while quantitative proteomics is still fully developing. In the present work microsomal proteins of *Medicago truncatula* roots were, for the first time, scrutinised by the state-of-the-art gel-free proteomic approach iTRAQ-OGE-LC-MS/MS. Herein, we described a straightforward, robust, and iTRAQ compatible method for in-solution protein digestion. Besides, the practical applicability of this tailored workflow allows users to successfully employ it with different kind of matrices. Another positive aspect of the current work includes the power of peptide fractionation according to their pI in OGE with respect to the focusing quality observed in free and iTRAQ labelled peptide electrophoresis.

Although Ross and co-workers described minimal iTRAQ reactivity with tyrosyl residue side-chain (<3%) [4], a recent study has reported an evidence of O-acylation of hydroxylated side-chains of amino acid residues with iTRAQ especially in positions near histidyl residue [33]. Herein, the examination of the peptide electrofocusing behaviour before and after iTRAQ labelling revealed a non-negligible basic pI shift in OGE fractionation on a wide pH range 3-10 and an important increase in retention time in LC separation of labelled peptides focused in OGE fraction 1. It was furthermore found that this basic shift is not global, specific peptides, with specific sequence-determined properties, may even have a shift to a more acidic pI. In this study, a first effort was done to describe these properties. It is noteworthy to point out that to date, most pI calculator algorithms use only native peptide sequences without taking into account the iTRAQ tags. Consequently, further experiments in combination with trustworthier, advanced pI calculator software are crucial to enhance our understanding on the observed basic shift and routinely describe the pI of iTRAQ labelled peptides. Thus, the experimental isoelectric points can be used as an efficient additional filtering tool for the validation of peptide identifications and increase the reliability of the identification procedure.

## Methods

### Biological material and growth conditions

*Medicago truncatula* cv Jemalong 5 seeds were surface sterilised and germinated at 27°C in the dark on 0.7% sterile agar [34]. Two-day old seedlings were then transplanted into 400 mL plastic pots containing a mix of sterile soil of Epoisses and sand (1:2 v/v). Mycorrhizal inoculation was realized by adding Epoisses soil-based inoculum (spores, roots and hyphae) of the AM fungus *Rhizophagus irregularis* DAOM 181602 (formerly known as *Glomus intraradices)*[35] . Seedlings (3 per pot) were grown for 4 weeks under controlled conditions (16 h photoperiod, 23°C/18°C day/night, 60% relative humidity, 220 μEinstein m^-2^.s^-1^ photon flux density). Control and *R. irregularis*-inoculated plants were watered each day with demineralised water and twice a week with a nitrogen-enriched nutrient solution. At harvest, roots were removed from their substrate, gently rinsed with deionised water, deep frozen, and stored at -80°C for later protein extraction.

### Microsomal protein extraction

Microsome extraction of *M. truncatula* roots was performed at 4°C and obtained by differential centrifugation as previously described by Stanislas and co-authors [21]. Briefly, roots were homogenized using a Waring Blendor in grinding buffer (50 mM Tris-MES, pH 8.0, 500 mM sucrose, 20 mM EDTA, 10 mM DTT and 1 mM PMSF). The homogenate was centrifuged at 16,000x*g* for 20 minutes (rotor JA 14 Beckman, CA, USA). After centrifugation, supernatants were collected, filtered through two successive meshes (63 and 38 μm), and centrifuged at 96,000x*g* for 1 h (rotor 45 Ti, Beckman). Pellets, representing the microsomal fraction, were resuspended in 10 mM Tris-MES, pH 7.3 250 mM sucrose, 1 mM EDTA, 1 mM DTT, 1 mM PMSF, 10 μg/ml aprotinin and 10 μg/ml leupeptin. Protein amount was measured using the 2-D Quant Kit (GE Healthcare, Little Chalfont, UK).

### In-solution protein digestion

Amicon Ultra-4 10 K centrifugal devices (Millipore, Bedford, MA, USA) were used for in-filter protein digestion. In the filtration devices, proteins (50 μg) from each control and mycorrhized plants were mixed with 10 mM DTT in solution A (8 M urea in 0.1 M Tris–HCl, pH 8.5) for 20 minutes to reduce protein disulphide bonds, and the excess reducing solution eliminated by centrifugation at 5,000x*g* for 40 minutes (Sanyo MSE Harrier 18/80, Japan). The protein sample was further cleaned by rinsing with 200 μl of solution A and repetition of the centrifugation step at 5,000x*g* for 40 minutes. One hundred microliters of 50 mM iodoacetamide in solution A were added on the filter and the filter was incubated in the dark at room temperature (RT) for 30 minutes followed by a centrifugation at 5,000x*g* for 30 minutes. Afterwards, 100 μl of solution B (8 M urea in 0.1 M Tris–HCl, pH 8.0) was added on the filter to adjust the pH, and centrifuged again. After repeating the latter step twice, trypsin (Trypsin Gold, mass spectrometry grade, Promega, Madison, WI, USA) was added in 30 μl of triethylammonium bicarbonate (TEAB) to an enzyme/protein ratio of 1:100. Protein digestion was carried out overnight at RT. Finally the peptides were collected by centrifugation of the filter units at 5,000x*g* for 40 minutes.

### iTRAQ peptides labelling

Each tryptic digest was either labelled with iTRAQ reagent 114 or 117 following the manufacturer's instructions (AB SCIEX, Foster City, CA, USA). Control and mycorrhized samples were labelled by alternating 114 and 117 tags to avoid labelling bias. Some experiments were labelled with single use of one iTRAQ tag (114, 115, 116 and 117) targeting an overview of the iTRAQ 4-plex effect on peptide pI. iTRAQ reagents were dissolved in 70 μl of ethanol and added to the protein digest. After 1 h of incubation at RT, equal amounts of the different samples were pooled and concentrated by evaporation using a SpeedVac (Heto, Saskatoon, SK, Canada). The excess of iTRAQ reagents was removed by desalting the labelled peptides using C18 columns Supelco (Discovery^TM^ DSC-18, 1 ml, 100 mg, Supelco Bellefonte, PA, USA). Peptides were eluted in 50% ACN (v/v), 0.1% TFA (v/v) and subsequently dried in SpeedVac (Heto) prior to peptides OGE fractionation.

### Peptide OGE

3100 OFFGEL Fractionator and OFFGEL Kit pH 3–10 (Agilent Technologies, CA, USA) with 12 wells setup were used. Peptides were diluted in 1.8 ml of the focusing buffer containing only 5% (v/v) of glycerol in deviation from the supplier’s protocol. IPG strips were rehydrated by adding 40 μl of peptide IPG strip rehydration solution per well for 15 minutes. Then, 150 μl of sample were loaded in each well. Peptide focusing was performed until it reached 20 kVh with a maximum voltage of 8,000 V and maximum current of 50 μA. After focusing, the 12 peptide fractions were withdrawn and wells rinsed with 150 μl of H_2_O/MeOH/TFA (49/50/1 v/v) for 15 minutes. Rinsing solutions were pooled with their corresponding peptide fractions and concentrated in SpeedVac (Heto) prior to LC-MS/MS analysis.

### LC-MS/MS analysis

The dried peptides were re-dissolved in 25 μl 0.1% TFA (v/v). Peptide separation was performed using an Ultimate 3000 nano LC system (Dionex, Sunnyvale, USA) equipped with a C18 column (PepMap 100, 3 μm, 100 Å, 75 μm id x 15 cm, Dionex) and connected to a Probot microfraction collector (Dionex). The mobile phase consisted of a gradient of solvents A 2% ACN (v/v), 0.2% TFA (v/v) in water and B 80% ACN (v/v), 0.08% TFA (v/v) in water. Peptides were separated at a flow rate of 0.3 μL/minute using a linear gradient of 60 minutes of solvent B from 0 to 5% in 5 minutes, followed by an increase to 30% in 5 minutes and to 65% in 30 minutes. The column was washed with 95% of solvent B for 5 minutes followed by regeneration with solvent A. Column effluent was mixed with MALDI matrix α-cyano-4-hydroxycinnamic acid (CHCA) and collected at a frequency of one spot every 30 seconds on an Opti-TOF LC/MALDI insert blank plate (AB SCIEX). MALDI plates were analyzed with a MALDI-TOF/TOF 4800 Proteomics Analyzer (AB SCIEX). The instrument was calibrated using the 4700 mass standard calibration kit (AB SCIEX). MS spectra between m/z 900 and 4,000 were acquired for every spot using 1,500 laser shots. The 8 most intense ion signals per spot having a S/N > 30 were selected as precursors for MS/MS acquisition.

Peptide and protein identifications were performed with the ProteinPilot™ Software 4.0.8085 revision 148085 (AB SCIEX) using the Paragon algorithm. Combined data and spectra from each OGE fraction were searched against the NCBI viridiplantae database (released on the 5^th^ of May 2011) and a EST database of *M. truncatula* (http://www.medicago.org, released on the 18^th^ of July 2011). The following search parameters were selected: iTRAQ 4-plex peptide label, cysteine alkylation, trypsin specificity, ID focus on biological modifications, and processing including quantitation and thorough ID. We only report protein identifications with a total ProtScore >1.3, which represents >95% statistical confidence in ProteinPilot. Proteins having at least one peptide above 95% of confidence were recorded.

MarvinSketch Calculator Plugin (http://www.chemaxon.com/marvin/sketch) [16], was implemented in this study to overcome the main bottleneck of the current available pI calculator such as the pI/MW tool of the ExPASy Proteomic Server (http://www.expasy.org) not giving the opportunity to calculate the pI of chemically modified peptides and consequently the pI of iTRAQ labelled peptides. This tool has been used to calculate pI of unlabelled and iTRAQ labelled peptides to explain some experimentally observed pI shifts.

## Abbreviations

ACN, Acetonitrile; AM, Arbuscular mycorrhiza; CHCA, α-cyano-4-hydroxycinnamic acid; DTT, Dithiothreitol; EDTA, Ethylenediaminetetraacetic acid; IAA, 2-iodoacetamide; IEF, Isoelectric focusing; IPG, Immobilized pH gradient; iTRAQ, Isobaric tags for relative and absolute quantitation; LC, Liquid chromatography; MALDI-TOF, Matrix assisted laser desorption ionisation time of flight; MeOH, Methanol; MES, 2-(N-morpholino)ethanesulfonic acid; MS, Mass spectrometry; MW, Molecular weight; NMWL, Nominal molecular weight limit; OGE, OFFGEL electrophoresis; pI, Isoelectric point; PMSF, Phenylmethylsulfonyl fluoride; SCX, Strong-cation exchange; TEAB, Triethylammonium bicarbonate; TFA, Trifluoroacetic acid; Tris, Tris(hydroxymethyl)aminomethane; 2-DE, Two-dimensional gel electrophoresis.

## Competing interests

The authors declare that they have no competing interests.

## Authors’ contributions

CA: has performed the experiment in the frame of her PhD thesis, from plant cultivation until the in-depth analysis of the data; KS: provided help for MS analysis, discussed the results and critically commented and worked on the manuscript; CG: helped for the plant material, and the microsome isolation; EDG: supervised the PhD work and critically commented the manuscript; CL: technical help and support, especially in liquid chromatography steps; JR: supervised the PhD work, discussed the results and critically commented and worked on the manuscript. All authors read and approved the final manuscript.

## Supplementary Material

Additional file 1 **Impact of iTRAQ labelling on peptide pI and OGE fractionwise.** This table shows the reproducibility of observed peptide pI shifts in at least 3 independent experiments of labelled samples in groups A, B, C and D. It presents peptide sequences, Paragon confidence score, observed (Obs mass) and theoretical masses (Theo mass), the mass modification induced by iTRAQ labelling and OGE fractions (OGF) before and after the labelling. Moreover the retention time (Rt) of non-modified and labelled peptides together with the difference (Diff) in retention time due to the iTRAQ tags are as well shown in this table. Click here for file

Additional file 2 **Impact of iTRAQ labelling on peptide retention time in LC separation.** This table presents the 17 peptides that showed an increase in retention time after the labelling. These peptides were focused in OGE fraction 1 when non-labelled (NL) and shifted to more basic fractions after iTRAQ labelling (L) and. The table shows their retention time (Rt) in minutes in LC separation before and after the labelling. Click here for file
